# Basis Cranii Interna in Metopism: A Comparative Geometric Morphometric Study

**DOI:** 10.3390/biology15010036

**Published:** 2025-12-25

**Authors:** Silviya Nikolova, Diana Toneva

**Affiliations:** Department of Anthropology and Anatomy, Institute of Experimental Morphology, Pathology and Anthropology with Museum, Bulgarian Academy of Sciences, 1113 Sofia, Bulgaria; ditoneva@abv.bg

**Keywords:** persistent metopic suture, internal cranial base, µCT, dry crania, geometric morphometrics

## Abstract

The cranium is a highly integrated structure composed of partially independent yet interrelated modules with different origin and developmental pathways. Throughout growth, these modules interact extensively in response to various intrinsic and extrinsic factors, ultimately forming a cohesive and functionally unified structure. Consequently, morphological changes in one cranial unit are expected to influence the development and shape of others. Considering the known vault alterations associated with metopism, we assume that the cranial base is also modified in metopic skulls. To test this hypothesis, we compared shape and size of the internal cranial base in metopic (46) and control (183) dry crania of contemporary adult Bulgarian males using geometric morphometric techniques. The crania were scanned using an industrial µCT system. Three-dimensional coordinates of 37 (9 midsagittal and 14 bilateral) landmarks were recorded on the endocranial surface. The landmarks were grouped into four configurations outlining the internal cranial base and its compartments: anterior cranial fossa, middle cranial fossa, and posterior cranial fossa. No significant size differences were observed between the metopic and control series. Shape comparisons revealed significant differences in all configurations except the posterior cranial fossa. However, Principal Component Analysis did not demonstrate clear separation between the groups, indicating that the observed shape variation cannot be attributed solely to the persistence of the metopic suture.

## Simple Summary

Metopism, defined as the persistence of the metopic suture into adulthood, represents a variation in cranial morphology. Although often considered an isolated feature of the frontal bone, its occurrence may reflect integrated developmental processes involving the cranial base. The cranial base plays a central role in coordinating craniofacial growth, serving as a structural and functional platform for the developing brain and facial skeleton. Consequently, variations in its growth pattern, synchondrosis activity, or overall geometry could influence the development of the entire craniofacial complex. This study explores the relationship between metopism and cranial base morphology by examining differences in shape and size using geometric morphometric approaches. Understanding this developmental relationship provides insight into both normal and variant cranial growth, with implications for developmental biology, clinical assessment, and the study of craniofacial integration.

## 1. Introduction

The cranium is a highly integrated structure composed of partially independent yet interrelated modules, which follow different origin and developmental pathways. It comprises the neurocranium, which includes the cranial vault and the cranial base, and the splanchnocranium. The cranial vault and cranial base follow different ossification patterns: the vault forms by intramembranous ossification, whereas the base develops via endochondral ossification. Similarly to the cranial vault, the splanchnocranium develops by intramembranous ossification. In terms of cellular origin, the anterior and lateral regions of the vault, including the frontal bones and squamous parts of the temporal bones, along with many facial bones and the anterior section of the cranial base, derive from neural-crest cells. The posterior segments of the vault and base, including the parietal bones, occipital bone, and petrous portion of the temporal bones, originate from paraxial mesoderm [1]. However, during growth these separate units interact extensively in response to various intrinsic and extrinsic stimuli, and gradually form the highly integrated cranial complex [2,3,4].

The neurocranium expands in response to the growing brain demands. Sutures and synchondroses serve as active growth centers for new bone formation through apposition and remodeling. Basically, the sutures and synchondroses oriented in the coronal plane allow antero-posterior expansion of the neurocranium, while those oriented in the sagittal plane permit medio-lateral widening [2]. The metopic suture is one of the major calvarial sutures, located between the two halves of the frontal bone, and is the only calvarial suture that closes by the end of the second postnatal year [5]. In some individuals, the metopic suture remains open into adulthood, a condition known as metopism, which occurs at varying frequencies across populations [6]. While the metopic suture remains patent, it allows continued widening of the frontal part of the vault. Consequently, an unusually prolonged metopic suture function is naturally linked with a specific frontal bone morphology. Although the factors that delay or prevent normal closure of the metopic suture remain uncertain [7], numerous morphological modifications in skulls with metopism have been documented. The major and more obvious one is the wider, shorter and more protruding occipital squama in the midsagittal plane [8,9]. Due to this reason, most of the studies have been focused on comparative investigations of the vault. Thus, in addition to the distinctive features of the frontal bone, a range of other significant morphological differences have been documented in metopic skulls. For example, these include modification in the shape and size of vault segments, delayed closure of the sagittal suture, underdevelopment of the frontal sinus, and an increased incidence of supernumerary calvarial bones [9,10,11]. Despite the focus on vault changes, little is known about modifications in the cranial base in metopism. Thus, except for some assertions and a few comparative investigations [12,13], the cranial base morphology in metopism still remains largely unknown. Since the cranial base is the platform upon which the rest of the skull grows and attaches, it is considered a structural foundation of the craniofacial architecture. It is also the first region of the skull to reach adult size and provides the foramina for nerves and vessels through which the brain communicates with the rest of the body [3]. Thus, even subtle changes in the cranial base geometry can influence the vault morphology, how facial structures align, and how neurovascular channels are oriented. That is why it is important to understand if there are any specific modifications of the cranial base in metopism as well.

Geometric morphometrics (GM) offers techniques for investigation of shape and size of biological structures represented as configurations of landmarks [14,15]. In previous investigations, the growing brain has been considered a major factor controlling cranial size, and less involved as a controlling force producing cranial form [16], while growth of the bones of the cranial base has been shown to be an important factor determining the overall shape of the skull [3]. Since the cranium is an integrated structure, it is expected that during the development and growth, modifications in one module (e.g., the vault) would impact another (e.g., the base). Given the known vault alterations in metopism, our hypothesis is that the cranial base in metopic skulls would also be modified. In this study we used GM approaches to compare the shape and size of cranial base in metopic and control cranial series.

## 2. Material and Methods

### 2.1. Material

For the purpose of the study osteological material preserved in the Military Mausoleum with Ossuary at the National Museum of Military History of Bulgaria was used. The stored skeletal remains belonged to Bulgarian soldiers who participated in the wars at the beginning of the 20th century. From the available material, a series of 229 (183 control and 46 metopic) adult male dry crania was selected. The investigation was approved by the Human Research Ethics Committee of the Institute of Experimental Morphology, Pathology and Anthropology with Museum at the Bulgarian Academy of Sciences (Protocol No 7/30.10.2018).

### 2.2. Methods

#### 2.2.1. Data Collection

The selected crania were scanned with an industrial µCT system Nikon XT H 225 (Nikon Metrology, Tokyo, Japan) according to the previously elaborated optimal scanning protocol: a voltage of 100 kV, a power of 10 W, a tube current of 100 µA, an exposure time of 500 ms. Under these conditions was acquired a series of 2000 sequential projections. CT Pro 3D (Nikon Metrology, Tokyo, Japan) was used for image reconstruction. The voxels were isotropic and the spatial resolution was 97.549 μm. Postprocessing of the images was performed following a previously elaborated protocol for image stack merging [17].

**Figure 1:**
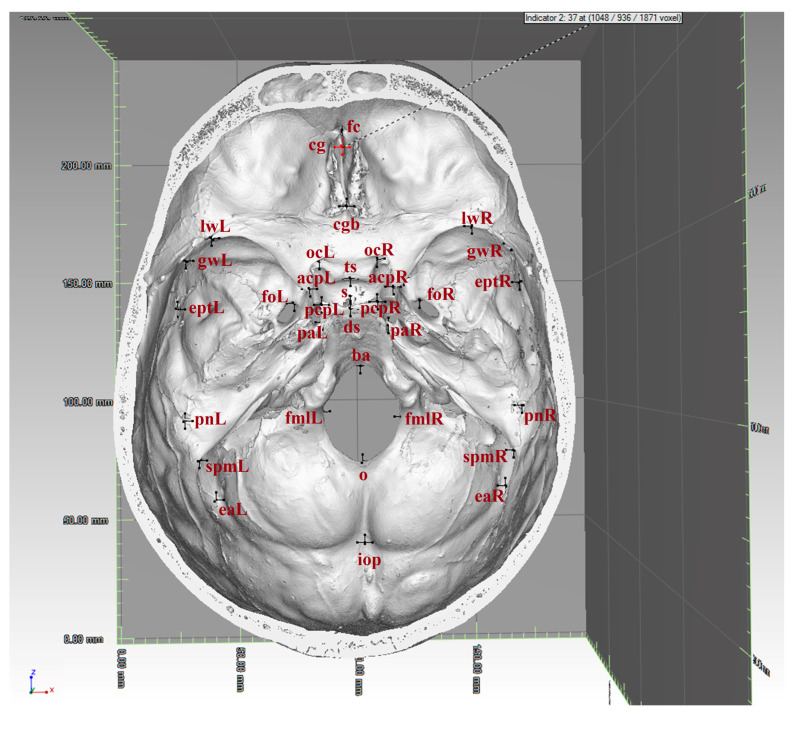
Endocranial surface with picked landmarks (for landmark abbreviations see Table 1).

The 3D coordinates of 37 (9 midsagittal and 14 bilateral) landmarks were collected on the endocranial surface (Figure 1, Table 1) using the “Clipping box” and “Indicator” tools in VG Studio Max 2.2. The landmarks were grouped into four configurations outlining the internal cranial base (ICB) and its compartments: anterior cranial fossa (ACF), middle cranial fossa (MCF), and posterior cranial fossa (PCF) (Table 2).

#### 2.2.2. Data Analyses

Intraobserver measurement error

The intraobserver error was assessed by three separate trials of the landmarks digitization on 30 crania conducted by one observer on separate days. The error of digitization for each landmark was assessed in millimeters based on the landmark standard deviation according to von Cramon-Taubadel et al. [18]. The error was computed based on the Euclidian distances of the repeated placement of the landmark to the landmark centroid. The landmark centroid was calculated for each specimen by averaging the 3D coordinates from the different trials of digitization of that landmark. The average digitation error for each landmark was used for assessment of the intraobserver precision in landmark digitization. The interobserver error was evaluated according to Lagravère et al. [19] as follows: acceptable, values within 1 mm; useful, values between 1 and 2 mm; and inappropriate for further analyses, values of more than 2 mm.

Geometric Morphometrics

Centroid size (CS) represents the square root of the sum of squared distances between the landmarks, included in the landmark configuration, and their centroid. CS was used as a measure of the landmark configuration size. The normal distribution of the CS values in each series was evaluated by Shapiro-Wilks normality test. The intergroup comparison of the CS for each landmark configuration was performed using the independent *t*-test.

Generalized Procrustes superimposition (GPS) was applied to the raw coordinates of each landmark configuration. The symmetric component of the obtained Procrustes coordinates was used in the analyses. To reduce the size-related differences in shape (allometry) a multivariate regression between the Procrustes coordinates and log-transformed CS was performed (number of randomization rounds: 10,000).

For dimensionality reduction and exploration of the shape variation in the sample was applied Principal component analysis (PCA). As a result, a smaller number of uncorrelated variables (principal components, PCs) were produced and were used in further analysis.

The multivariate normal distribution of the shape data was evaluated by Mardia’s multivariate skewness and kurtosis tests [20] and the Doornik and Hansen omnibus test [21], and due to the non-normal distribution, the shape variables (regression residuals) of the two series were compared by one-way PERMANOVA on PCs accounting for 95% of shape variance (permutations: 10,000 iterations; Euclidean distance measure).

The statistical analyses were performed in MorphoJ 1.07a [22] and Past 4.17c [23].

## 3. Results

### 3.1. Intraobserver Error of Landmark Digitization

**Figure 2:**
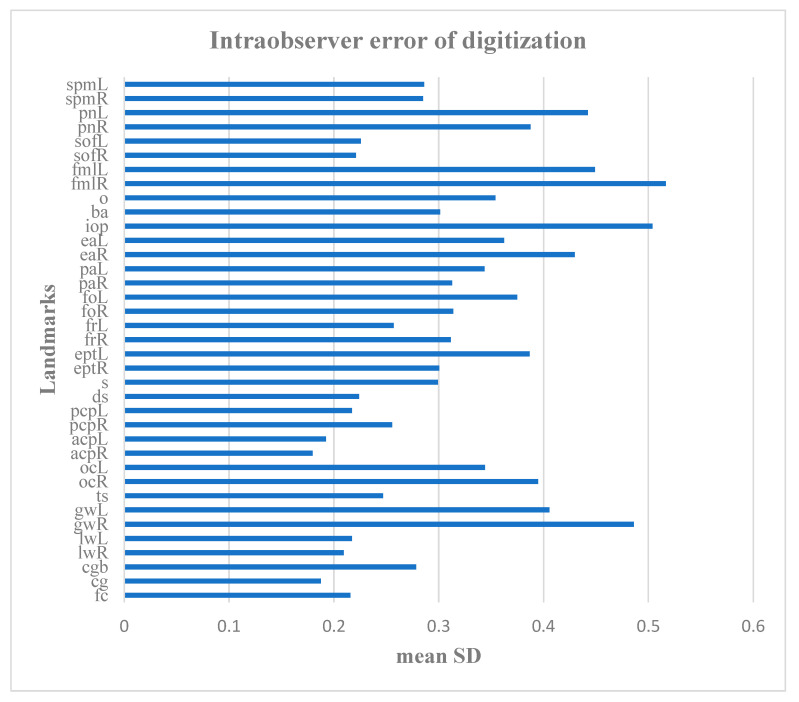
Intraobserver error of landmark digitization in millimeters (for landmark abbreviations see Table 1).

The error of digitization was acceptable since it was less than 0.6 mm for all of the landmarks (Figure 2).

### 3.2. Size

The comparison did not show any significant size differences in the landmark configurations between the series (Table 3).

### 3.3. Allometry

The regression of the Procrustes coordinates on CS showed a significant impact of size on shape (Table 4). The size of the ICB, MCF and PCF configurations accounted for a relatively small percentage of the shape variation, whereas the percentage of the shape variation explained by size for the ACF was higher in both series. Therefore, to eliminate the allometry effect, the regression residuals were used as an input shape data for PCA and intergroup comparisons.

### 3.4. Shape

**Figure 3:**
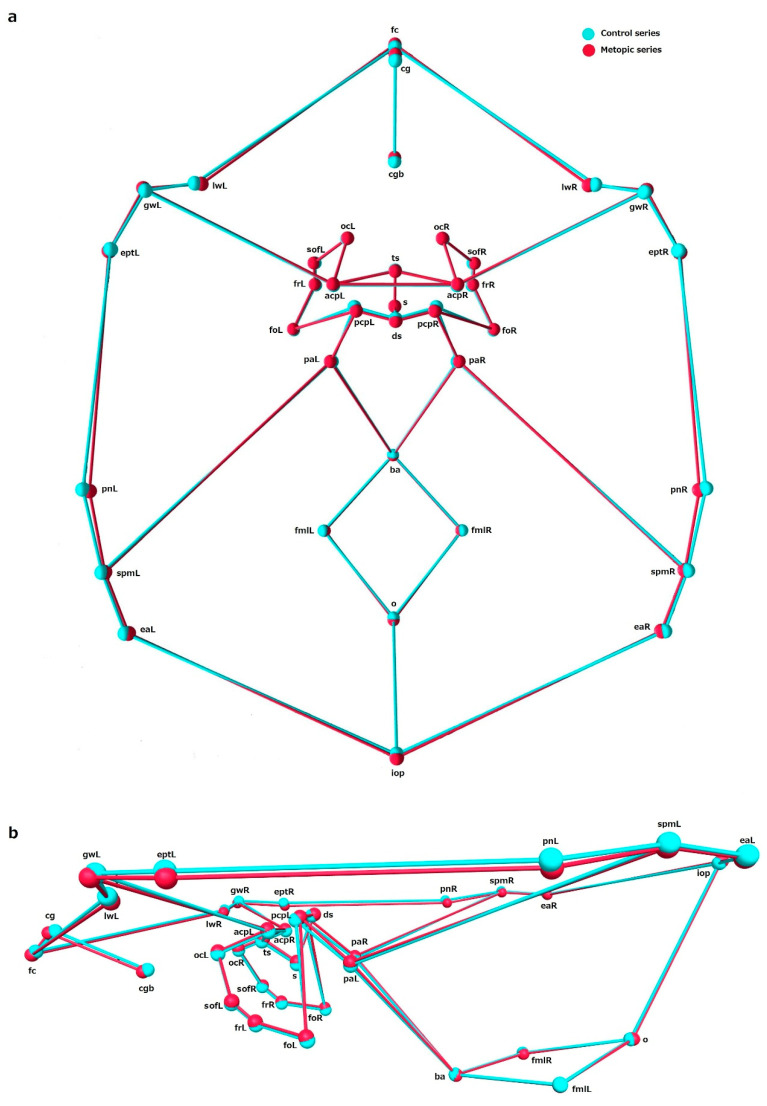
The mean landmark configurations of the internal cranial base in the control and metopic series in: (**a**) superior view and (**b**) lateral view.

**Figure 4:**
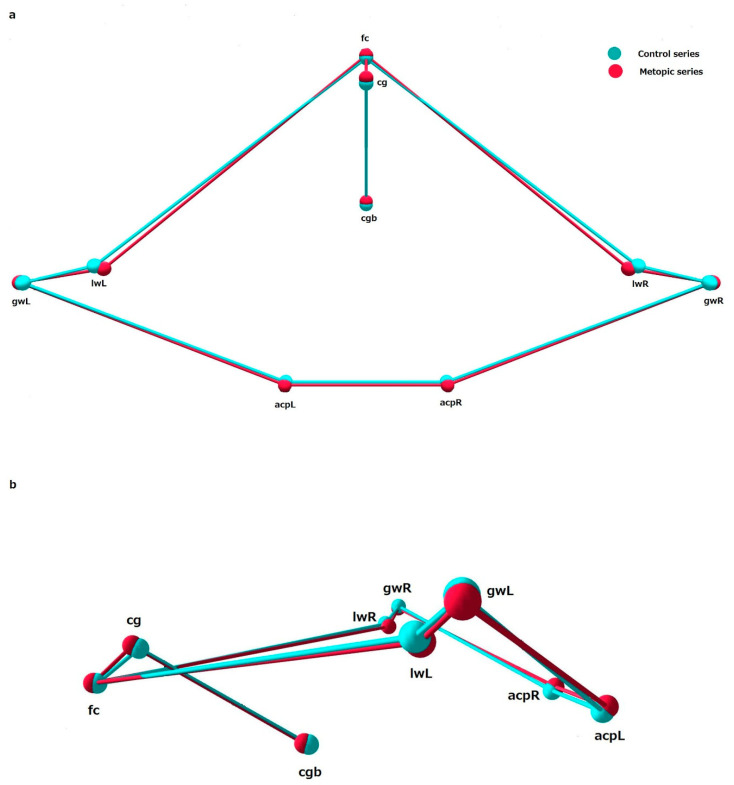
The mean landmark configurations of the anterior cranial fossa in the control and metopic series in: (**a**) superior view and (**b**) lateral view.

**Figure 5:**
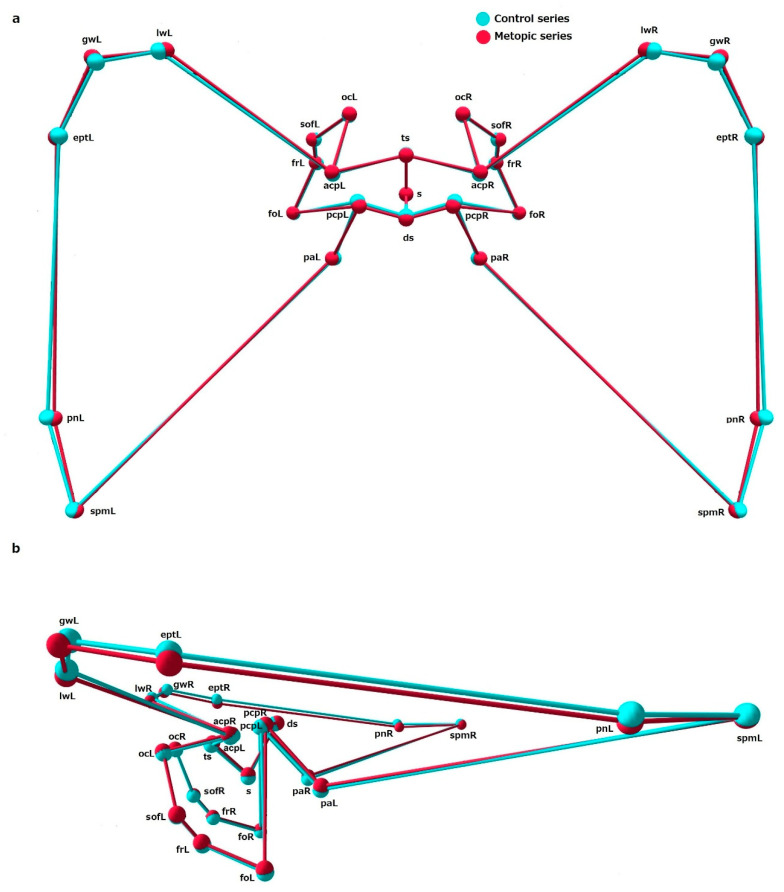
The mean landmark configurations of the middle cranial fossa in the control and metopic series in: (**a**) superior view and (**b**) lateral view.

**Figure 6:**
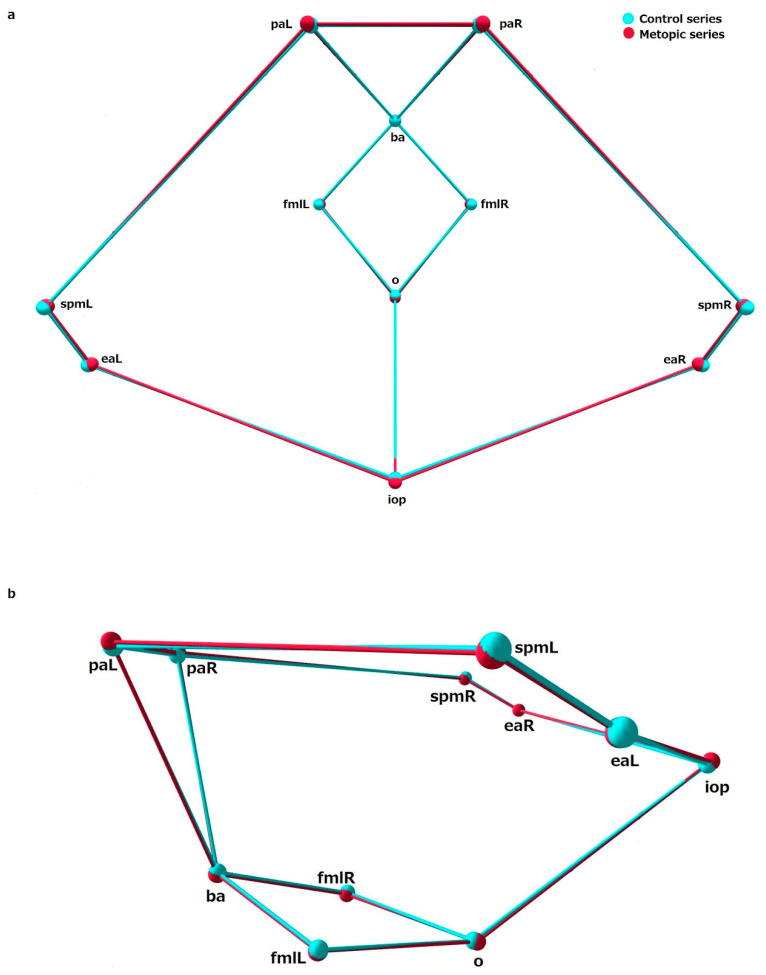
The mean landmark configurations of the posterior cranial fossa in the control and metopic series in: (**a**) superior view and (**b**) lateral view.

The comparison between the shape variables in the metopic and control series showed significant differences in shape of ICB, ACF and MCF, while the PCF did not differ in shape between the series (Table 5). Analyzing the mean coordinates of the landmarks across configurations allows the main shape differences between the series to be identified (Figure 3, Figure 4, Figure 5 and Figure 6).

ICB: In the metopic series, the landmarks demarking the central part of the cranial base, around the sphenoid body, were placed superiorly compared to the same landmarks in the control. All other landmarks delineating the lateral contours of the cranial base and the foramen magnum in metopic crania were in an inferior position compared to the landmarks in the control. In the metopic series, the anterior midsagittal landmarks (fc, cg, cgb) were more anteriorly placed, while the midsagittal landmarks in the middle (ts, ds) and posterior (ba, o, iop) partitions were more posteriorly placed. The landmark sella was an exception since in both series its position was overlapping; although, in the metopic series it was placed in a slightly more superior position (Figure 3). This tendency could be seen in detail in each of the separate configurations of the cranial fossae.

ACF: In the metopic series, the landmarks foramen cecum (fc), crista galli (cg) and crista galli base (cgb) were placed more anteriorly, compared to these landmarks in the control. The landmarks on the lesser wings (lwR and lwL) in the metopic crania were placed in a more medio-inferior position compared to the control. The landmarks designating the position of the great wings on both sides (gwR and gwL) in the metopic crania were placed slightly more laterally and inferiorly to these landmarks in the control. The mean coordinates of the landmarks on the anterior clinoid processes in the metopic series on both sides (acpR and acpL) were slightly superio-posteriorly placed compared to the same landmarks in the control (Figure 4).

MCF: In the metopic series, most of the landmarks in the central part of the sphenoid bone, the sphenoid body and foramina (s, ts, acpR-L, ocR-L, sofR-L, frR-L, foR-L, paR-L), were placed superiorly to the same landmarks in the control, except for the landmarks outlining the posterior border of sella turcica (pcpR-L, ds), which were located in a slightly anterosuperior position compared to the corresponding landmarks in the metopic series. The landmarks demarking the lateral borders of the MCF (lwR-L, gwR-L, eptR-L, pnR-L, spmR-L) in the metopic series were placed slightly inferiorly to the corresponding landmarks in the control (Figure 5).

PCF: In the metopic series the landmarks around the foramen magnum (ba, fmlR-L, o) were placed inferiorly to these landmarks in the control (Figure 6).

### 3.5. Principal Component Analysis

**Figure 7:**
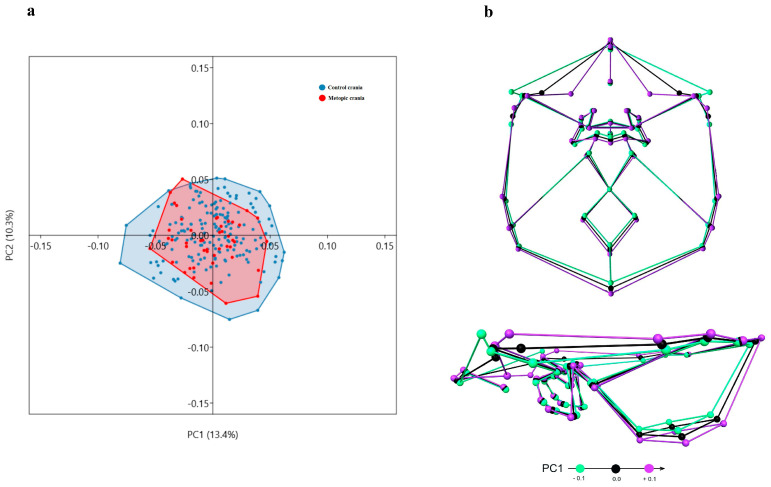
Variation in shape of the internal cranial base: (**a**) distribution of the skulls by PC1 and PC2; (**b**) superior and lateral view of the shape variation on PC1.

**Figure 8:**
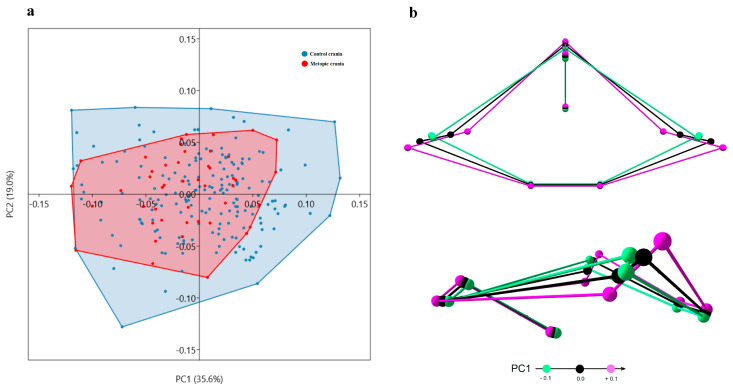
Variation in shape of the anterior cranial fossa: (**a**) distribution of the skulls by PC1 and PC2; (**b**) superior and lateral view of the shape variation on PC1.

**Figure 9:**
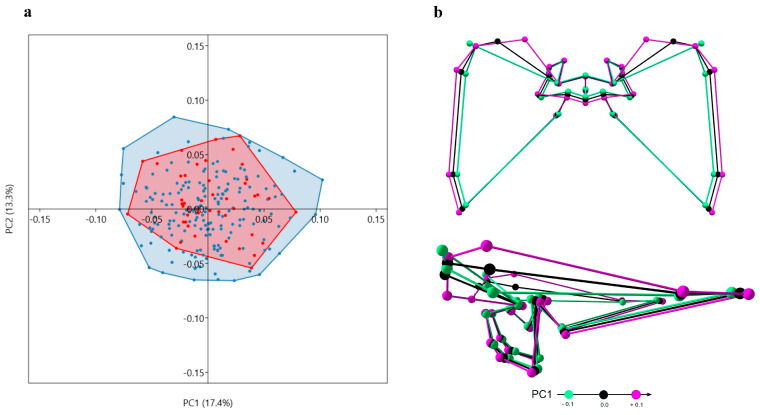
Variation in shape of the middle cranial fossa: (**a**) distribution of the skulls by PC1 and PC2; (**b**) superior and lateral view of the shape variation on PC1.

**Figure 10:**
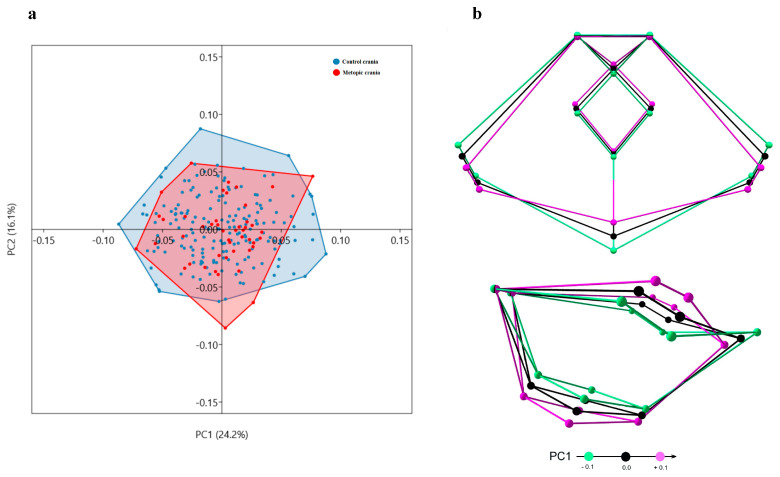
Variation in shape of the posterior cranial fossa: (**a**) distribution of the skulls by PC1 and PC2; (**b**) superior and lateral view of the shape variation on PC1.

PCA did not indicate a considerable separation between the metopic and control series along the axes of the individual principal components (PC). Furthermore, the main PCs (PC1 and PC2) accounted for a relatively small percent of the total variation in the sample (Figure 7, Figure 8, Figure 9 and Figure 10); i.e., the variation was not related to a particular vector. Rather, it was dispersed.

ICB: In the sample, the shape of the ICB varies on PC1 from a relatively wider ACF with more lateral and closely located lesser and greater sphenoid wings, shallow MCF and PCF with an inferior position of the landmarks outlining the lateral borders of the MCF and a superior position of foramen magnum in the PCF, to a shape with a narrow ACF and deeper MCF and PCF (Figure 7). This tendency, along with some specific shape modifications, could be seen in more details in the landmark configurations of each cranial fossa (Figure 8, Figure 9 and Figure 10).

ACF: According to PC1, the ACF shape in the sample varies from a wider anterior (fc-lw) and a narrow posterior (gw-acp) lateral borders with closely placed lesser (lw) and greater (gw) sphenoid wings, and more posteriorly placed midsagittal landmarks (fc, cg, cgb), to a shape with narrow anterior and wider posterior lateral borders, more remotely placed lesser and greater sphenoid wings and more anteriorly located midsagittal landmarks (Figure 8).

MCF: The shape of the MCF varies from a wider one with more medially placed lesser wings to a narrow shape with more closely located lesser and greater sphenoid wings (Figure 9).

PCF: On PC1, the shape of the PCF varies from a relatively shallow PCF with more posteriorly placed landmarks delineating foramen magnum and the interior occipital protuberance, to a deeper and shorter PCF (Figure 10).

## 4. Discussion

In normal development, cranial sutures serve as functional growth interfaces: they permit the displacement and expansion of adjacent bone plates in response to internal demands of the growing brain [24,25]. Meanwhile, the cranial base contributes to overall skull lengthening through growth at synchondroses, which resemble growth plates in many respects [2,3]. Morphometric studies have shown that in skulls with persistent metopic suture, the frontal region often undergoes mediolateral expansion, along with a shortening in the anteroposterior direction and a protrusion in the midsagittal plane [9]. These changes are consistent with the extended growth potential in the frontal region, since the metopic suture remains active long enough to permit additional lateral expansion, beyond the normal closure period. The modification of the frontal part of the vault in metopism naturally raises the question whether the cranial base changes as well. Findings in the present study have indicated a lack of size differences in the cranial base at all, but significant shape differences in all configurations except for the PCF. Recent comparative investigation of the cranial base in metopism have also demonstrated that certain distances in the anterior and middle cranial fossae are significantly longer in metopic skulls, whereas the transverse diameters do not differ significantly compared to the controls [13]. These findings indicate that the cranial base in metopism is also modified, but in a manner that does not fully mirror the vault changes. Thus, while the vault undergoes expansion through widening, the base does not exhibit equivalent changes, which suggests that alterations in one module (e.g., the vault) are not directly translated to the other (the base). This could be related to the different growth of the vault and base considering the ossification mechanisms, timeframes and growth drivers. In metopism, the vault is directly impacted by the persistent metopic suture and demonstrates specific changes, while the base, which matures earlier, shows subtler modifications that do not exactly reflect those of the vault. Nevertheless, although the adjustments in the vault and base in metopism do not mirror each other perfectly, similar to the vault, the significant changes in the base, both in shape and in some antero-posterior dimensions, are located in the anterior and middle segments.

In metopism, the sustained metopic suture activity may modify how forces generated by brain growth are distributed within the vault and transferred to the base. From the perspectives of Moss’s functional matrix theory, skeletal units (bones, sutures, cartilage) grow not on their own initiative but in response to the functional demands of the soft tissue matrices (brain, dura, soft tissues) they enclose or support [24]. In this context, the persistent metopic suture may reflect prolonged or altered functional matrix activity in the frontal brain region in response to continued soft tissue growth demands. The dural reflections and fibrous attachments from cranial base to vault designate future suture locations and mediate mechanotransductive signaling between brain and vault [24]. Because cranial sutures coincide with dural insertions and zones of mechanical tension, their patency depends on osteogenic signals from the underlying dura. Prolonged suture persistence may therefore reflect delayed or reduced dural-derived growth stimuli, altered mechanical tension, or both [25]. The vault, under continued functional demand, expands laterally, while the base, growing under a different matrix regime and ossification mode, responds more conservatively or selectively. Consequently, in metopism the vault reflects direct response to extended functional matrix pressure, while the base reacts in more constrained ways, producing partial but not parallel morphologic shifts. So, metopism should not be considered simply as an anatomical variation; rather, it demonstrates how cranial modules respond differentially to altered growth regimes.

Metopism is correlated with subtle but measurable deviations in cranial shape and size, an increased incidence of supernumerary calvarial bones and delayed closure of the sagittal suture [10,11,13]. So, the persistent metopic suture does not seem to be an isolated anatomical variation. Rather, the condition “metopism” could be interpreted as a possible reflection of mild or partial alterations in the signaling pathways controlling ossification. Cranial vault and base development are coordinated by a network of common and region-specific signaling pathways that regulate osteogenesis and endochondral growth [4]. Both regions share core regulatory mechanisms, including FGF/FGFR, BMP, Wnt/β-catenin, and TGF-β signaling, which modulate osteoblast and chondrocyte differentiation and coordinate overall craniofacial growth [26]. Despite these shared pathways, the vault and cranial base differ in their modes of ossification and region-specific signaling requirements. The cranial vault develops through intramembranous ossification, and suture patency is particularly sensitive to gradients of FGF and Wnt activity that regulate osteoblast proliferation, as well as transcriptional regulators such as RUNX2 and TWIST1 [25]. In contrast, the cranial base grows via endochondral ossification, where the IHH–PTHrP feedback mechanism plays a key role in maintaining proliferative chondrocytes, controlling hypertrophy, and timing synchondrosis fusion [27,28]. Additional modulators such as BMP and Wnt/β-catenin guide the transition from cartilage growth to ossification, while systemic factors like the GH/IGF-1 axis influence matrix production and overall growth dynamics [29]. All these pathways are highly dose-dependent, such that even mild imbalances can alter ossification patterns without necessarily producing clinically significant alterations or syndromes.

Since the cranium is an integrated unit, even mild dysregulation in osteogenic signaling is expected to influence both vault and base development. A shift toward reduced osteogenic activity, through decreased Wnt/β-catenin, BMP, or related signaling, would affect both modules in a region-specific manner. In the vault, reduced osteogenic signaling would decrease osteoblast proliferation across sutures, potentially altering suture morphology and delaying closure [25]. In the cranial base, diminished signaling would slow the transition from proliferative to hypertrophic chondrocytes in the synchondroses, delay synchondrosis fusion and endochondral ossification [27,28]. This would result in prolonged antero-posterior growth, retention of larger cartilaginous zones, and delayed establishment of cranial base angles. A delayed synchondrosis maturation and fusion corresponds to the significantly enlarged antero-posterior dimensions of the base in metopic skulls, as the changes are located in ACF and MCF [13]. The ACF enlarges primarily through growth at the spheno-ethmoidal synchondrosis. The mid-sphenoid synchondrosis also contributes partially to the ACF elongation, whereas the spheno-occipital synchondrosis, mainly influence MCF and PCF rather than ACF [2,3]. Thus, in metopism the anterior synchondroses of the base seem to be more affected than the posterior ones. Nonetheless, the base angulation in metopism does not appear to be altered [12].

Studies in animal models, particularly mice, demonstrate that cranial base growth plays a central role in shaping cranial vault morphology. Experimental perturbations of basicranial synchondrosis growth alter vault geometry and redistribute mechanical strain across cranial sutures, consequently influencing suture patency and timing of fusion [4,30]. Consistent with this, morphometric analyses show that variation in the sphenoid and other basicranial elements covaries with vault curvature and height, indicating that synchondrosis growth can drive coordinated changes in vault form [31,32]. In humans, the vault–base integration is amplified by rapid postnatal brain growth, with increased basicranial flexion closely linked to vault expansion and globularity during development [33,34,35]. Considering the distinctive cranial morphology of metopic crania, metopism could be interpreted not merely as a localized suture variation, but as an expression of coordinated cranial development, in which cranial base growth, vault formation, and brain expansion interact to alter the timing of metopic suture closure.

## 5. Conclusions

The obtained results showed that there were no significant size differences in the landmark configurations between the control and the metopic crania. Except for PCF, all other configurations demonstrated significant shape differences between the series. PCA did not show clear separation between the investigated series for any of the landmark configurations, which indicated that the observed shape variation in the sample could not be explained solely by the persistence of the metopic suture. Thus, in metopism, calvaria, the upper segment of the neurocranium and its compartments, differs in shape and size, while the cranial base, i.e., the lower segment and its subunits, differs in shape, but not in size.

## Figures and Tables

**Table 1:** Description of the landmarks [13].

Landmark	Description
Midsagittal	
Basion (ba)	A point on the anterior margin of foramen magnum in the midsagittal plane
Crista galli (cg)	The most prominent point on crista galli
Dorsum sellae (ds)	A point at dorsum sellae in the midsagittal plane
Crista galli base (cgb)	A point placed at the posterior ridge of crista galli in its base
Foramen cecum (fc)	A point on the posterior margin of foramen cecum in the midsagittal plane
Internal occipital protuberance (iop)	A point on the internal occipital protuberance
Opisthion (o)	A point on the posterior margin of foramen magnum in the midsagittal plane
Sella (s)	A point at the center of sella turcica in the midsagittal plane
Tuberculum sellae (ts)	A point on the tuberculum sellae in the midsagittal plane
Bilateral	
Anterior clinoid process (acp)	The most prominent point on the anterior clinoid process
Endoasterion (ea)	The point of intersection of the lambdoid, occipitomastoid and parietomastoid sutures on the endocranial surface
Endopterion (ept)	The meeting point between the greater wing of the sphenoid, the parietal bone and the temporal squama on the endocranial surface
Foramen magnum laterale (fml)	The most lateral point on the margin of foramen magnum
Foramen ovale (fo)	The most medial point on foramen ovale on the endocranial surface
Foramen rotundum (fr)	A point on the medial ridge of foramen rotundum
Greater wing of the sphenoid (gw)	The meeting point between the greater wing of the sphenoid, the parietal bone and the frontal bone on the endocranial surface
Superior orbital fissure (sof)	The most inferior point on the superior orbital fissure on the endocranial surface
Lesser wing of the sphenoid (lw)	The sharpest point at the site of the articulation with the frontal bone
Optic canal (oc)	A point on the medial ridge of the optic canal
Posterior clinoid process (pcp)	The most prominent point on the posterior clinoid process
Parietal notch (pn)	The point of intersection of the squamous suture and the parietomastoid suture on the endocranial surface
Petrous apex (pa)	A point at the apex of the petrous part of the temporal bone
Superior petrous margin (spm)	A point at the intersection of the superior margin of the petrous part of the temporal bone, formed between the anterior and posterior surfaces, with the parietomastoid suture, on the endocranial surface

**Table 2:** Description of the investigated landmark configurations.

Landmark Configuration		Landmarks			
		T	M	B	Abbreviations
1	Internal cranial base	37	9	14	All investigated landmarks
2	Anterior cranial fossa	9	3	3	fc, cg, cgb, lwR, gwR, acpR, acpL, gwL, lwL
3	Middle cranial fossa	27	3	12	ts, s, ds, acpR, ocR, iofR, frR, foR, pcpR, paR, spmR, pnR, erpR, gwR, lwR, acpL, ocL, iofL, frL, foL, pcpL, paL, spmL, pnL, erpL, gwL, lwL,
4	Posterior cranial fossa	11	3	4	ba, o, iop, eaR, fmlR, spmR, paR, paL, spmL, eaL, fmlL

T—total number of landmarks; M—midsagittal landmarks; B—bilateral landmarks.

**Table 3:** Centroid size of the landmark configurations and comparison between the two series.

Landmark Configuration	Control		MS		Differences	
	Mean	SD	Mean	SD	*t*-Test	*p*-Value
Cranial base	292.83	8.23	293.24	8.75	t = 0.304	0.761
Anterior fossa	105.45	6.16	105.64	6.48	t = 0.186	0.853
Middle fossa	213.84	7.86	213.38	8.85	t = 0.341	0.733
Posterior fossa	147.34	4.24	146.93	4.56	t = 0.576	0.566

**Table 4:** Regression of the Procrustes coordinates on centroid size.

Landmark Configuration	Total		Control		MS	
	% *	*p*-Value	%	*p*-Value	%	*p*-Value
Cranial base	2.39%	<0.0001	1.84%	0.0002	7.49%	<0.0001
Anterior fossa	17.50%	<0.0001	17.53%	<0.0001	19.71%	<0.0001
Middle fossa	3.06%	<0.0001	2.56%	0.0001	6.76%	0.0002
Posterior fossa	1.79%	<0.0001	1.12%	0.0467	8.28%	0.0001

*—percent of predicted variation.

**Table 5:** Shape differences assessed by one-way PERMANOVA on PCs accounting for 95% of shape variance.

Landmark Configuration	Total Sum of Squares	Within-Group Sum of Squares	F	*p*-Values
Cranial base (PC1–PC34)	1.176	1.166	1.966	0.014
Anterior fossa (PC1–PC9)	1.554	1.534	2.835	0.019
Middle fossa (PC1–PC23)	1.466	1.453	2.055	0.021
Posterior fossa (PC1–PC11)	1.010	1.006	0.723	0.651

## Data Availability

The data presented in this study are available on request from the corresponding author due to ethical reasons.
